# Cytoplasmic domain of CM2 is involved in the replication of influenza C virus

**DOI:** 10.1099/jgv.0.002165

**Published:** 2025-10-17

**Authors:** Yoshitaka Shimotai, Kanetsu Sugawara, Yoko Matsuzaki, Yasushi Muraki, Ri Sho, Takanari Goto, Hiroshi Hamamoto, Seiji Hongo

**Affiliations:** 1 Department of Infectious Diseases, Yamagata University Faculty of Medicine, Yamagata, Japan; 2 Division of Infectious Diseases and Immunology, Department of Microbiology, School of Medicine, Iwate Medical University, Iwate, Japan; 3 Department of Public Health and Hygiene, Yamagata University Graduate School of Medical Science, Yamagata, Japan

**Keywords:** CM2, cytoplasmic domain, genome packaging, influenza C virus, viral replication, virus uncoating

## Abstract

CM2 is a single-pass transmembrane protein located in the viral envelope and is essential for the growth of influenza C virus. We previously reported that CM2 is involved in viral genome packaging and uncoating. We also demonstrated that alanine substitutions at residues 47–48, 67–69, 73–75, 85–87 and 113–115 in the cytoplasmic domain of CM2 significantly reduced its expression levels. Herein, we analysed whether these mutations affected viral replication. The alanine-substituted recombinant viruses rCM2-Ala67–69, 73–75 and 85–87 exhibited significantly reduced replication compared with recombinant wild-type viruses (rWT), and the amount of CM2 protein in virions and infected cells was also reduced compared with that in rWT. In addition, the amount of viral RNA within the particles of these mutant viruses was significantly lower than that in rWT. Furthermore, the amount of viral RNA-NP complexes transported into the nucleus of infected cells was reduced in rCM2-Ala73–75 and 85–87 viruses. In contrast, recombinant viruses with mutations at positions 113–115 could not be rescued, and virus-like particles containing this mutation showed suppressed genome packaging and uncoating and reduced expression of the CM2 protein. These results suggested that the cytoplasmic region of CM2 contributes to efficient packaging and uncoating of the genome through its stable expression.

**Impact Statement**

The CM2 protein of the influenza C virus plays a vital role in viral replication, yet the precise contribution of its cytoplasmic domain remains elusive. In this study, we identified key amino acid residues within this domain that are essential for genome packaging and uncoating and further demonstrated that these regions also influence CM2 protein stability and intracellular trafficking. These findings provide new insights into the functional organization of CM2 and contribute to a broader understanding of the molecular features associated with viral replication. This work will be of particular interest to researchers investigating virus assembly, host–virus interactions and the biology of enveloped RNA viruses.

## Introduction

Influenza C virus (ICV) is a member of the family *Orthomyxoviridae* and possesses a genome composed of seven segments of negative-sense ssRNA [1]. ICV primarily causes mild upper respiratory tract infections in children but can occasionally lead to more severe illnesses, such as pneumonia and bronchitis, particularly in children under 2 years of age and in patients with underlying medical conditions [2,3]. ICV encodes multiple proteins essential for replication, including NP, HE, M1, CM2, three polymerase subunits (PB1, PB2 and P3) and nonstructural proteins (NS1 and NS2). The RNA segment 6 (M gene) encodes the matrix (M1) and CM2 proteins, which consist of 242 and 115 amino acids, respectively [4].

The M2 protein of the influenza A virus (IAV) is a CM2 homologous protein that has proton ion channel activity and is responsible for transporting protons from the endosome to the viral interior. This process disrupts the interaction between vRNA and M1, thereby promoting viral uncoating [5]. The M2 protein is also required for efficient packaging of vRNA into viral particles and is involved in the production of infectious virions and viral morphogenesis. Furthermore, deletion or alanine substitution of the cytoplasmic domain of the M2 protein has been reported to reduce infectious IAV production and affect virion morphology, suggesting its importance for virion structure and vRNA packaging [6,8]. We previously demonstrated that CM2 is involved in the packaging of reporter genes into virus-like particles (VLPs) and for VLP uncoating, indicating that CM2 is essential for viral replication [9].

CM2 is a single-pass transmembrane protein consisting of a 23-amino-acid N-terminal extracellular domain, a 23-amino-acid transmembrane domain and a 69-amino-acid C-terminal cytoplasmic domain [10,11]. The extracellular region of CM2 contains N-glycosylation (occurring in three forms: unglycosylated CM2_0_, high-mannose-type glycosylated CM2a and complex-type glycosylated CM2b) and disulphide bond formation sites that are involved in viral replication [12,13]; however, the cytoplasmic region remains poorly understood. In our previous study, we found that alanine substitution of the phosphorylated serine at position 78 (Ser78) significantly reduced the replicative capacity of the virus [14]. Furthermore, to investigate the amino acid regions within the cytoplasmic domain that affect the biochemical properties of CM2, mutant proteins carrying alanine substitutions were expressed. We identified amino acid regions involved in the expression levels, N-glycosylation and tetramer formation of the CM2 protein [15]. In particular, alanine substitutions at residues 47–48, 67–69, 73–75, 85–87 and 113–115 within the cytoplasmic domain significantly reduced CM2 protein expression levels.

In this study, we generated recombinant ICVs to analyse the effects of these amino acid sequences on viral replication. The results showed that recombinant viruses with mutant proteins of the above sequences suppressed replication, CM2 protein expression levels, genome packaging and uncoating to a greater extent than the WT viruses. Thus, we concluded that the cytoplasmic region of the CM2 protein is important for the stable expression of CM2, thereby maintaining efficient viral replication.

## Methods

### Cells and antibodies

293T, HMV-II and MDCK cells were cultured in Dulbecco’s modified Eagle’s medium, RPMI 1640 medium and Eagle’s minimal essential medium (MEM) supplemented with 10% FCS. Cells were cultured in an incubator at 37 °C in the presence of 5% CO_2_. Monoclonal antibodies against influenza C/Ann Arbor/1/50 HE (S16) [16], NP (H27) [17], M1 (L2) [18] and anti-CM2 serum [19] were used as previously reported.

### Construction of plasmids

Plasmids expressing segment 6 (M gene) RNA carrying alanine substitutions in the CM2 amino acid sequence, designated polI-CAA-M/CM2-Ala47–48, 67–69, 73–75, 85–87 and 113–115, and plasmids expressing mutant CM2 proteins, pME18S/Met-CM2-Ala47–48, 67–69, 73–75, 85–87 and 113–115, were constructed as follows: the plasmids polI-CAA-M [20] and pME18S/Met-CM2-YA [21] were used as templates, and the mutant sequences were amplified by inverse PCR using the primers shown in Table S1, available in the online Supplementary Material, and KOD-Plus-DNA polymerase (TOYOBO, Osaka, Japan). The full-length plasmid, including the mutated sequence, was amplified by inverse PCR using the primers listed in Table S1 and KOD-Plus-DNA polymerase. PCR products were self-ligated and transformed into *Escherichia coli*, followed by plasmid extraction after bacterial growth. The plasmids were sequenced using Sanger sequencing to confirm the introduction of the intended mutations. The amino acid sequence of the CM2 protein from ICV (C/Ann Arbour/1/1950) was obtained from GenBank (accession no. AB126196.2, protein ID: BAD24943.1)

### Generation of recombinant ICV

Recombinant wild-type influenza C virus (rWT) was generated using the reverse genetics method as previously described [22]. Recombinant viruses with mutant CM2, in which the amino acid sequences at positions 47–48, 67–69, 73–75, 85–87 and 113–115 of CM2 were substituted with alanine, were also generated using the same method and designated rCM2-Ala47–48, 67–69, 73–75, 85–87 and 113–115, respectively. Specifically, each mutant vRNA expression plasmid (polI-CAA-M/CM2-Ala47–48, 67–69, 73–75, 85–87 or 113–115) was used instead of the WT pPolI-CAA-M plasmid and cotransfected into 293T cells, along with the other six vRNA expression plasmids and nine protein expression plasmids. At 72 h post-transfection, the culture supernatant was harvested and inoculated into the amniotic cavity of 9-day-old embryonated chicken eggs to prepare viral stocks. The nucleotide sequence of the M gene in each recombinant virus was confirmed using Sanger sequencing.

### Virus growth and plaque assay

Each recombinant virus was used to infect MDCK cells at a multiplicity of infection (MOI) of 0.001 in the presence of 20 µg ml^−1^ of tosylamide phenylmethyl chloromethyl ketone (TPCK)-trypsin. Culture supernatants were collected at 0.5, 1, 2, 3, 4, 5 and 6 days post-infection and stored at −80 °C with glycerol to a final concentration of 10%.

The infectious titres of recombinant viruses in the supernatants were determined using a plaque assay as previously described [14]. Briefly, virus samples were serially diluted tenfold and inoculated into MDCK cells. After a 60 min incubation at 34 °C, the cells were overlaid with MEM containing 1% Avicel and 5 µg ml^−1^ TPCK-treated trypsin, followed by incubation at 34 °C for 4 days. The cells were fixed with 4% paraformaldehyde and permeabilized with 0.5% Triton X-100. Immunostaining was performed using monoclonal anti-NP antibody H27 as the primary antibody and HRP-conjugated goat anti-mouse IgG (H+L) as the secondary antibody. Plaques were visualized by staining with True Blue substrate (KPL, Gaithersburg, MD, USA) and counted [23].

### Detection of cell surface proteins

Viral proteins on the surface of infected cells were detected as previously described [12,13]. HMV-II cells infected with the recombinant virus were washed with ice-cold PBS 24 h post-infection and incubated with PBS containing 0.5 mg ml^−1^ sulfo-NHS-LC-biotin (Pierce, Rockford, IL, USA) for 30 min on ice. The cells were washed twice with PBS containing 100 mM glycine to stop the reaction, and RIPA buffer [0.01 M Tris–HCl (pH 7.4), 0.15 M NaCl, 1% sodium deoxycholate, 1% Triton X-100, 0.1% SDS] containing a protease inhibitor cocktail (Nacalai Tesque, Kyoto, Japan) was added and incubated on ice for 30 min to allow lysis. Cell lysates were centrifuged at 13,000 ***g*** for 20 min at 4 °C. The supernatant was mixed with a streptavidin-agarose bead resin (Pierce, Rockford, IL, USA) and incubated at 25 °C for 60 min. The resulting precipitates (biotinylated proteins) and total cell lysates were subjected to SDS-PAGE followed by Western blotting to detect HE and CM2 proteins.

### Immunoblotting analysis

Proteins extracted from recombinant viruses, purified VLPs and infected cells were separated using SDS-PAGE on a 17.5% polyacrylamide gel containing 4 M urea [14]. After SDS-PAGE, Western blotting was performed using monoclonal antibodies against HE (S16), NP (H27), M1 (L2) and anti-CM2 serum, as previously described [9,14]. Band intensities were measured using the ImageJ software ver. 1.54i (https://imagej.net/ij/).

CM2 oligomerization bands were detected as follows: first, recombinant viruses were solubilized under non-reducing conditions, and the samples were separated by SDS-PAGE as described above. Bands were detected by Western blotting with anti-CM2 serum. Protein signals were visualized using the Amersham ECL Prime Western blotting detection reagent (GE Healthcare, Chicago, IL, USA) with a LightCapture MG (ATTO) equipment. Band intensities were measured using the ImageJ software, and the relative amounts of CM2 dimers or tetramers per total lane intensity are shown in the graph.

### Real-time reverse transcription PCR (RT-PCR) analysis

Viral RNA (vRNA) from recombinant viruses was extracted from egg-derived viruses purified by ultracentrifugation using a sucrose cushion and an RNeasy Mini Kit (QIAGEN, Hilden, Germany). After DNA removal using TURBO DNA-free DNase (Ambion, Austin, TX, USA), cDNA was synthesized using a primer complementary to the conserved 12-nucleotide sequence present at the 3′ end of vRNAs [20]. Primer pairs specific for the NP gene of ICV, FLuCNP+1068 (5′-CGRTGTTCTGGGRCTTGCTTATG-3′) and FluCNP-1161 (5′-ARTTTTCCTATTTTCATTCTTGTTTCTCAAC-3′) and the FluCNP-1100 probe (5′-FAM-TTGGTTYTCTGCYATGGTYAGCCAYCCTCT-TAMRA-3′) [24] were used for real-time PCR. Real-time PCR was performed using the StepOnePlus Real-Time PCR System (Applied Biosystems, Foster City, CA, USA) and TaqMan Fast Universal PCR Master Mix (Applied Biosystems) as previously described [24]. A standard curve was generated based on a 10-fold serial dilution series of *in vitro* transcripts containing 10^2^–10^8^ copies of full-length NP vRNA.

### Virus entry assays

The viral stock used for this assay was prepared as follows. MDCK cells were infected with virus at an MOI of 0.01 and incubated with MEM containing 20 µg ml^−1^ TPCK-trypsin for 5 days at 34 °C. The culture was then centrifuged at 700 ***g*** for 5 min at 4 °C to collect the supernatant, which was concentrated through centrifugation at 4,000 ***g*** for 30 min at 4 °C using an Amicon Ultra-15 centrifugal filter device (Merck Millipore Ltd., molecular weight cutoff: 100 kDa). The concentrated virus solution was transferred to 1.5 ml tubes, mixed with glycerol to a final concentration of 10% and stored at −80 °C.

Recombinant viruses prepared as described above were inoculated into HMV-II cells at an MOI of 50 and incubated on ice for 30 min. The cells were then incubated with prewarmed medium at 34 °C for 60 or 120 min. After incubation, virus-infected cells were fixed with 4% paraformaldehyde for 15 min at 25 °C. Following fixation, the cells were permeabilized with PBS containing 0.2% Triton X-100 for 15 min at 25 °C. For immunostaining, a mouse monoclonal antibody YA27 against the NP protein, purified using protein A and diluted with CanGet Signal Immunostain solution B (TOYOBO, Osaka, Japan), was used as the primary antibody and incubated for 1 h at 25 °C. After washing, Alexa Fluor 488-conjugated goat anti-mouse IgG diluted in CanGet Signal Immunostain solution B was used as a secondary antibody and incubated for 1 h at 25 °C. Cell nuclei were stained with PBS containing 10 µg ml^−1^ Hoechst 33342 for 15 min at 25 °C. Microscopy was performed using an LSM700 confocal microscope (Carl Zeiss, Oberkochen, Germany) equipped with a 63× Plan-Apochromat oil imaging lens and 405 and 488 nm lasers. Cell images were analysed using the Zen Lite software ver. 3.5 (Carl Zeiss). To measure green fluorescence intensity in nuclei, nuclei with a diameter of ≥10 µm (Hoechst-stained regions) were selected, and 30–50 nuclei were measured per condition.

Endocytosis inhibition experiments were performed as follows. Cells were pretreated with RPMI 1640 medium containing 25 mM NH_4_Cl for 2 h before viral infection, and the rWT virus (MOI 50) was adsorbed onto HMV-II cells in the presence of 25 mM NH_4_Cl for 30 min on ice. After adsorption, the medium was then replaced with prewarmed RPMI 1640 medium containing 25 mM NH_4_Cl and incubated at 34 °C for 60 and 120 min. Virus-infected cells were fixed with 4% paraformaldehyde, immunostained with fluorescent antibodies, as described above, and analysed under a confocal laser microscope.

### Generation of VLP

WT-VLPs were generated by introducing a GFP-vRNA expression plasmid and nine viral protein expression plasmids into 293T cells, as previously described [20]. VLPs containing CM2 with substitutions at residues 113–115 (CM2-Ala113–115-VLP) were generated by introducing the CM2 protein expression plasmid pME18S/Met-CM2-Ala113–115 in place of pME18S/Met-CM2-YA. CM2-deficient VLPs (∆CM2-VLPs) were also prepared via transfection with the pME18S plasmid instead of pME18S/Met-CM2-YA. Culture supernatants were collected 48 h post-transfection, and VLPs were purified by ultracentrifugation using a 30% sucrose cushion. The resulting VLPs were suspended in PBS containing 10% glycerol and stored at −80 °C [9]. GFP-vRNA in the VLPs was extracted using the RNeasy Viral RNA Kit, as previously reported, and quantified by real-time RT-PCR [9].

### VLP infection of HMV-II cells and flow cytometry

Purified VLPs were treated with TPCK-trypsin (20 µg ml^−1^) for 10 min at 37 °C, and the reaction was stopped by adding soybean trypsin inhibitor. HMV-II cells were infected with equal amounts of VLPs with WT CM2 or mutant CM2 based on the amount of GFP-vRNA and incubated at 34 °C for 60 min. Subsequently, helper virus (C/Ann Arbor/1/50) was added at an MOI of 5 and incubated for 48 h. At 48 h post-infection, the cells were harvested using Accutase (Innovative Cell Technologies, San Diego, CA, USA) and fixed with 1% paraformaldehyde at 25 °C for 10 min. Fixed cells were suspended in PBS containing 3% FBS, and 100,000 cells per sample were analysed using a BD FACSCanto II flow cytometer (BD Biosciences, San Jose, CA, USA). Data were analysed using the FlowJo software version 10 (FlowJo, LLC, Ashland, OR, USA).

### Statistical analysis

Statistical analyses were performed using R version 4.2.2 (R Foundation for Statistical Computing, Vienna, Austria). The Student’s t-test was used to compare two groups, whereas one-way ANOVA followed by Tukey’s post-hoc test was used for comparisons among multiple groups. A P-value<0.05 was considered statistically significant and is shown in the figures as *P<0.05, **P<0.01 and ***P<0.001.

## Results

### Effect of mutations in the cytoplasmic region of CM2 on ICV replication

**Fig. 1.**
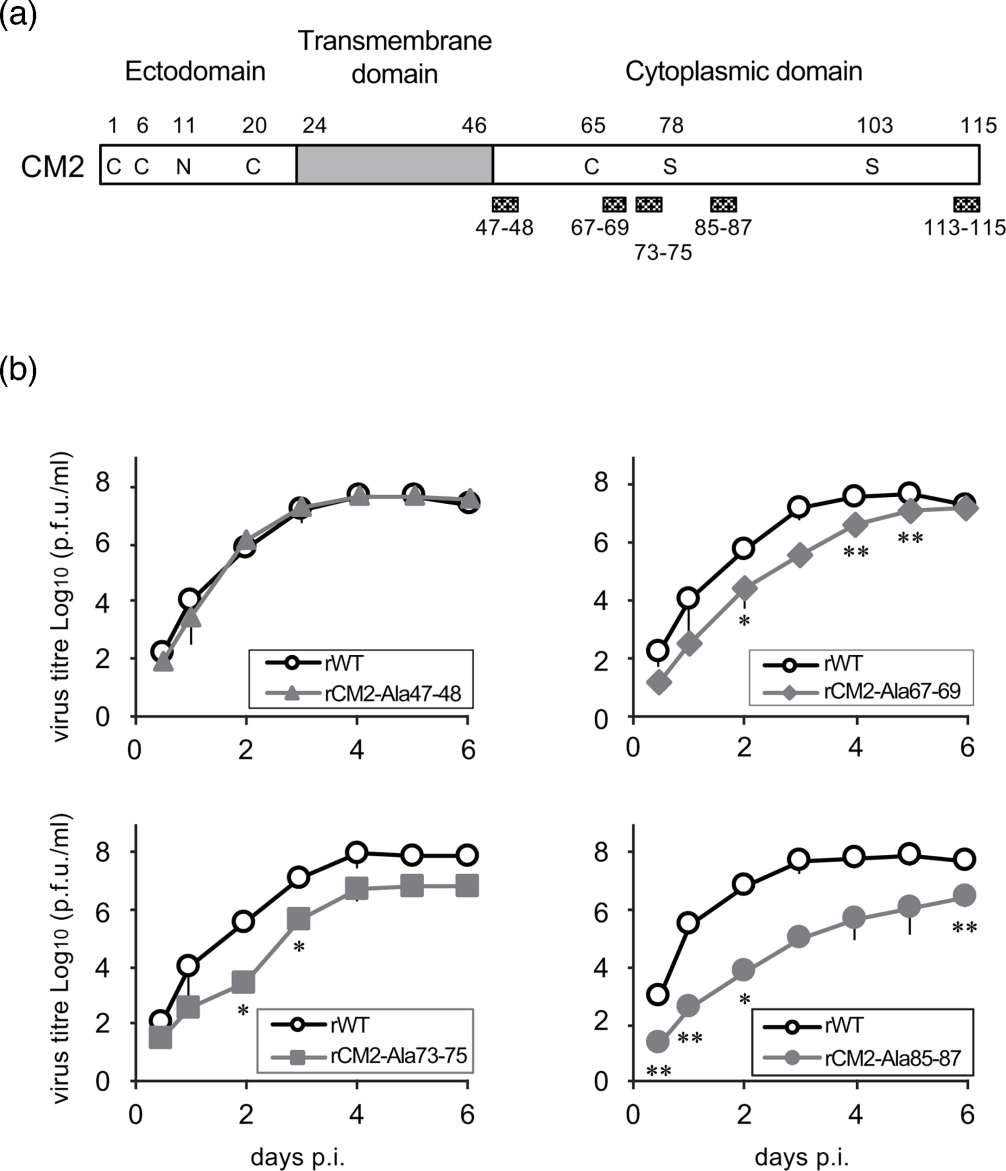
Growth curves of recombinant viruses carrying mutant CM2 proteins. (**a**) Schematic representation of CM2 mutation sites. Shaded boxes indicate the amino acid mutation sites (positions 47–48, 67–69, 73–75, 85–87 and 113–115) in the mutant CM2 protein. Among the cysteine residues, C1, C6 and C20 are involved in oligomerization, whereas C65 functions as a palmitoylation site. N and S indicate the sites of glycosylation and phosphorylation, respectively. (**b**) Growth curves of WT and mutant viruses. Statistical analysis was performed using Student’s t-test (*P<0.05; **P<0.01).

To investigate the effect of amino acid residues at positions 47–48, 67–69, 73–75, 85–87 and 113–115 of CM2 on ICV replication, we generated recombinant viruses in which these regions were substituted with alanine (Fig. 1a). The rCM2-Ala47–48, rCM2-Ala67–69, rCM2-Ala73–75 and rCM2-Ala85–87 viruses were successfully recovered, whereas the rCM2-Ala113–115 virus could not be rescued. These results suggested that the amino acid residues 113–115 of CM2 are essential for viral replication.

We compared the growth kinetics of the recovered mutant viruses with those of the WT virus (rWT). We found that the viral titres of rCM2-Ala47–48 were comparable to those of rWT, whereas those of rCM2-Ala67–69, 73–75 and 85–87 were significantly lower (Fig. 1b). Thus, these amino acid sequences are involved in the efficient replication of ICV.

### Expression of viral proteins in recombinant viruses and infected cells

**Fig. 2.**
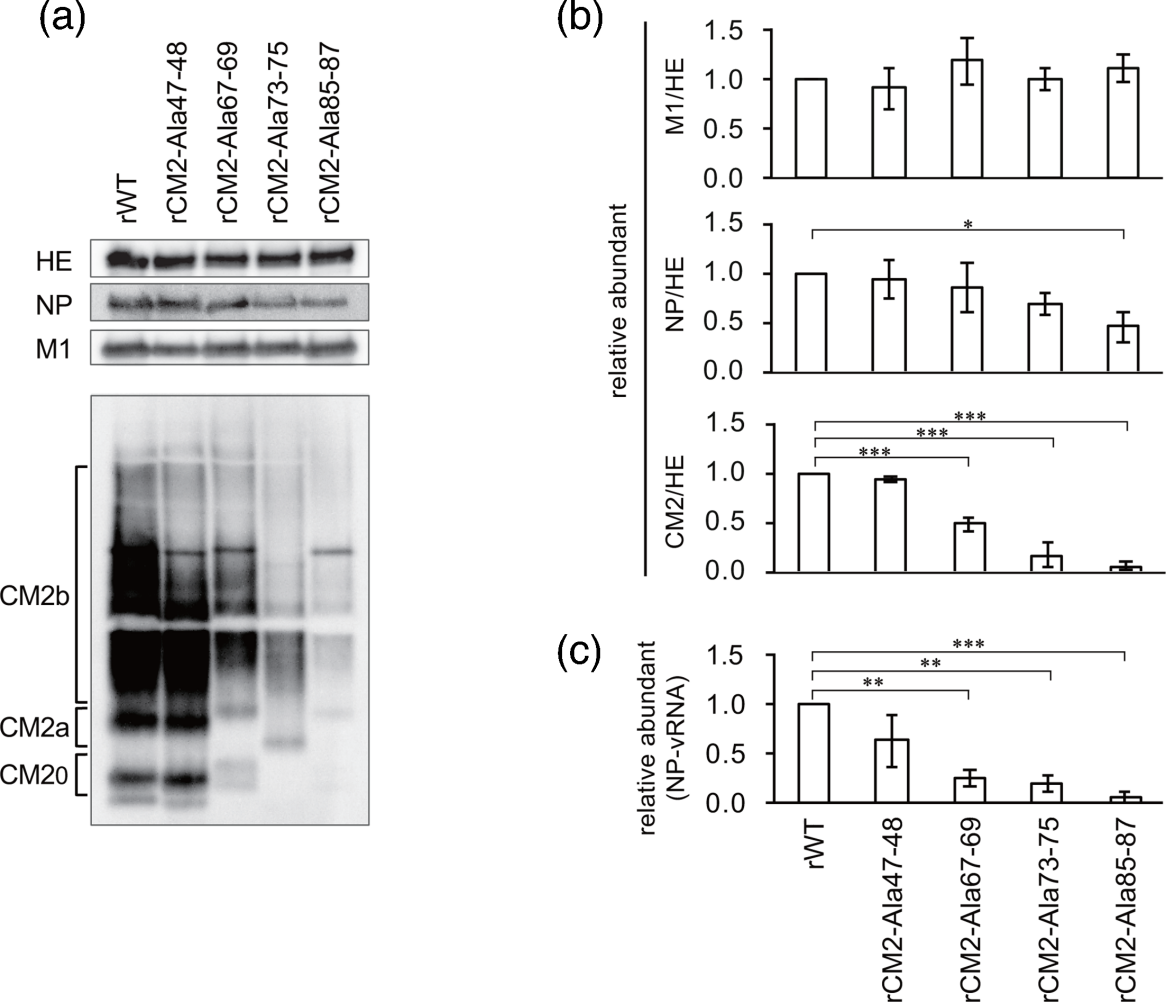
Analysis of viral protein expression and NP vRNA levels in recombinant virus particles. (**a**) Western blot analysis of HE, NP, M1 and CM2 proteins in recombinant virus particles. (**b**) Quantification of M1, NP and CM2 proteins in recombinant virus particles. Band intensities from (**a**) were measured in three independent experiments, and relative amounts of NP, M1 and CM2 protein were calculated using HE protein as a reference. The values were normalized to those of rWT. Statistical analysis was performed using one-way ANOVA followed by Tukey’s post-hoc test (*P<0.05, **P<0.01 and ***P<0.001). (**c**) Measurement of NP vRNA levels in recombinant virus particles. RNA was extracted from purified recombinant viruses, and NP vRNA levels were quantified by real-time RT-PCR. Copy numbers were normalized to those of rWT. Statistical analysis was performed as described in (**b**).

Our previous analysis showed that CM2 proteins with the abovementioned amino acid substitutions had reduced expression levels in cells compared with the WT protein [15]. Alanine substitutions in specific cytoplasmic regions of the M2 protein of IAV affect M1 protein incorporation and viral budding [6]. Therefore, we measured the amounts of HE, NP, M1 and CM2 proteins (hereafter referred to as protein levels) in these recombinant viruses to investigate any changes in the composition of structural proteins within the viral particles (Fig. 2a, b). We found that M1 protein levels did not differ from those of rWT for all viruses. In contrast, NP protein levels were significantly lower in rCM2-Ala85–87 than in rWT. CM2 protein levels in rCM2-Ala47–48 were comparable to those in rWT, whereas significant reductions were observed in rCM2-Ala67–69, 73–75 and 85–87 viruses. These results suggested that these regions of the CM2 protein are involved in protein expression and incorporation into virions. We previously reported that amino acid residues 47–48 and 73–75 in the cytoplasmic region of the CM2 protein are involved in N-glycosylation [15]. However, in the present study, we observed no differences in CM2 protein glycosylation among viruses (Fig. 2a). Therefore, the decrease in CM2 protein levels was likely not due to differences in glycosylation.

The amount of NP protein in virions is expected to reflect the amount of vRNA, as NP binds to vRNA at a stoichiometry of ~1 monomer per 24 nucleotides [25]. Therefore, to examine its effect on genomic packaging, we measured and compared the amount of segment 5 vRNA (encoding NP) in these virions using real-time RT-PCR. We found that the level of segment 5 vRNA in virions was significantly reduced in rCM2-Ala67–69, 73–75 and 85–87 viruses compared with that in rWT (Fig. 2c). These results suggested that sequences at positions 67–69, 73–75 and 85–87 of CM2 were involved in viral genome packaging.

**Fig. 3.**
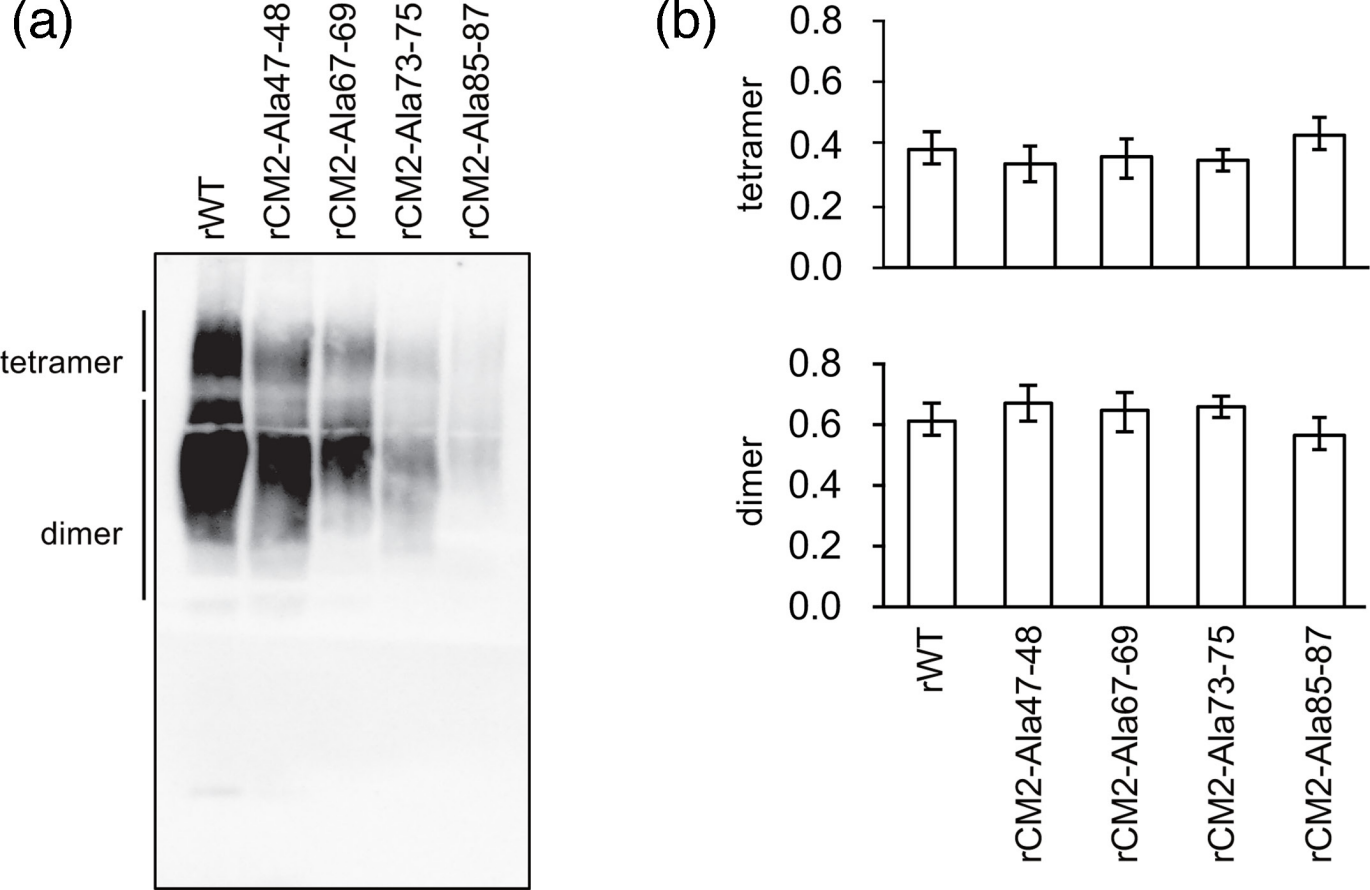
Comparison of CM2 oligomerization in recombinant virus particles. (**a**) Western blot analysis of CM2 protein in recombinant virus particles under non-reducing conditions. (**b**) Quantitative analysis of CM2 oligomerization in recombinant virus particles. Band intensities from (**a**) were measured in three independent experiments, and the amounts of CM2 dimers and tetramers were calculated relative to the total band intensity in each lane. Statistical analysis was performed using one-way ANOVA followed by Tukey’s post-hoc test; no significant differences were observed.

Furthermore, amino acid residues at positions 73–75 are involved in CM2 tetramer formation in cells [15]; however, whether this region affects CM2 multimerization in virions remains unclear. Therefore, we analysed the compositional ratios of dimeric and tetrameric CM2 proteins in virions. We observed that the ratio of dimeric and tetrameric CM2 was comparable to that of rWT (Fig. 3), indicating that tetramer formation was not reduced. Thus, these mutations did not impair the maturation from dimer to tetramer and had no effect on CM2 oligomerization in virions.

### Expression of CM2 protein on the cell surface of recombinant virus-infected cells

**Fig. 4.**
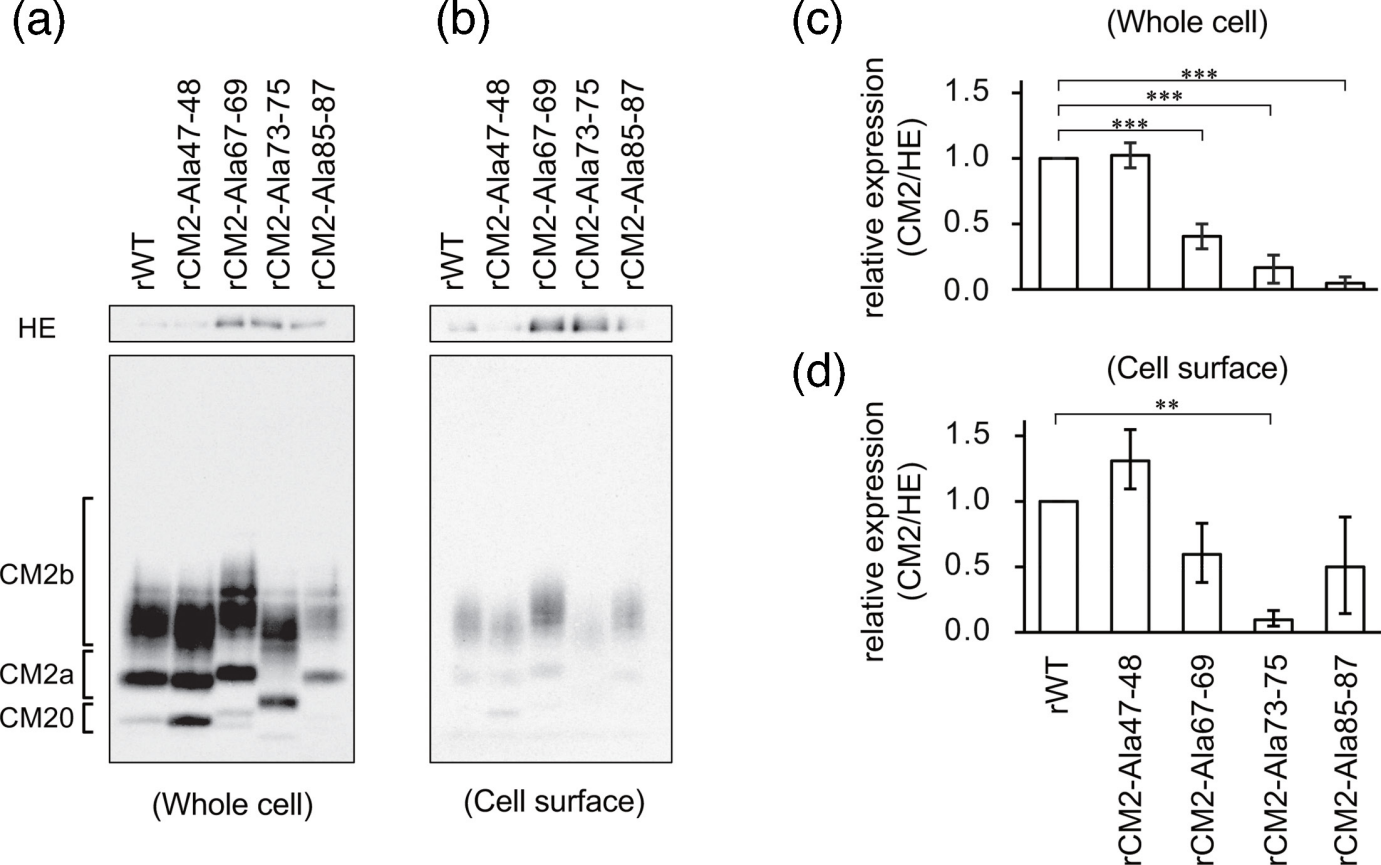
Expression levels of CM2 and HE proteins in virus-infected cells and on the cell surface. (**a**) Detection of CM2 and HE proteins in whole-cell lysates extracted from virus-infected cells. (**b**) Detection of CM2 and HE proteins present on the cell surface. (**c**) Quantification of CM2 protein expression in whole-cell lysates. Band intensities from (**a**) were measured in three independent experiments, and relative CM2 levels were calculated using HE protein as a reference. (**d**) Quantification of CM2 protein expression on the cell surface. The analysis was performed using the same method as in (**c**). In both (**c**) and (**d**), the CM2/HE ratio in rWT-infected cells was used as the normalization standard. Statistical analysis was performed using one-way ANOVA followed by Tukey’s post-hoc test (*P<0.05, **P<0.01 and ***P<0.001).

As shown in Fig. 2, the CM2 protein level in rCM2-Ala67–69, 73–75 and 85–87 viruses was lower than that in rWT. This may be attributed to reduced levels of the CM2 protein in virus-infected cells or at the cell surface where viral budding occurs. First, we examined the CM2 protein levels in virus-infected cells and found them to be significantly lower in cells infected with rCM2-Ala67–69, rCM2-Ala73–75 and rCM2-Ala85–87 viruses than in cells infected with rWT (Fig. 4a, c). Thus, these mutations reduce the intracellular expression of CM2. Next, we measured the CM2 protein levels expressed on the cell surface and found that surface expression was significantly reduced in rCM2-Ala 73–75-infected cells (Fig. 4b, d). These results suggested that the 73–75 region of the CM2 protein influenced its surface expression levels.

### Effects of amino acid sequences at positions 73–75 and 85–87 of CM2 protein on the uncoating process

**Fig. 5.**
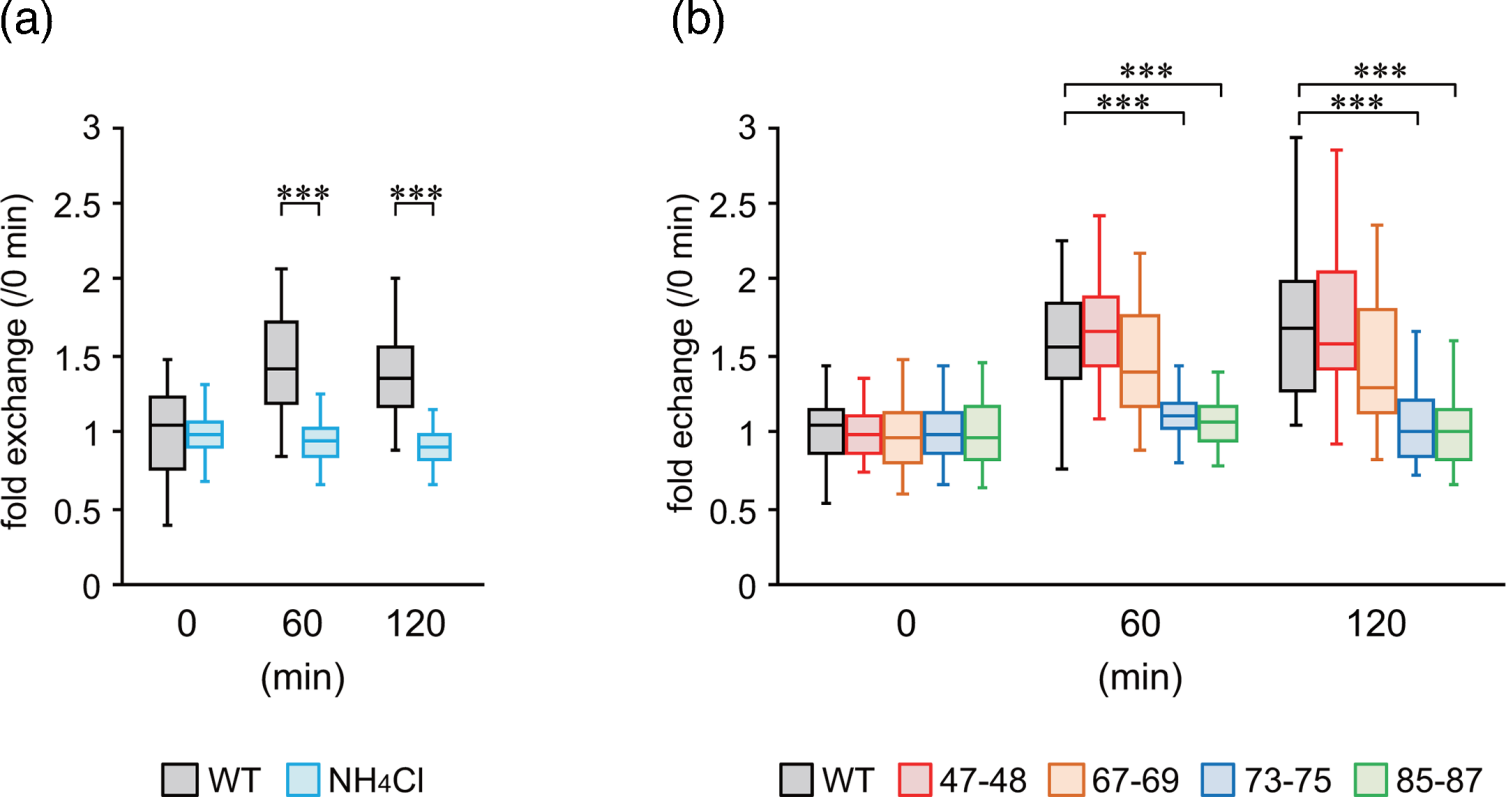
Nuclear localization of vRNPs (NP) during the early stage of viral infection. (**a**) Inhibition of vRNP nuclear import by ammonium chloride (NH_4_Cl) treatment. (**b**) Time course of nuclear localization of vRNPs relative to 0 min in cells infected with rWT or mutant viruses. For each recombinant virus, the value at 0 min was used for normalization. Representative results from two independent experiments are shown, with similar outcomes obtained in both. Statistical analysis was performed using one-way ANOVA followed by Tukey’s post-hoc test (*P<0.05, **P<0.01 and ***P<0.001).

We previously reported that CM2 is involved in the viral uncoating process [9]. After binding to cellular receptors, influenza viruses enter host cells via endocytosis. Acidification of the endosome triggers fusion between the endosomal membrane and viral envelope, leading to disassembly of the M1 layer and release of the viral genome into the cytoplasm, followed by its nuclear translocation. This process is dependent on a decrease in endosomal pH, and ammonium chloride (NH_4_Cl) inhibits uncoating by suppressing acidification [26]. Therefore, we first examined the localization of NP proteins in cells during the early stages of viral infection in the presence of NH_4_Cl, to confirm whether uncoating was inhibited under these conditions. Consequently, we found that in rWT-infected cells, nuclear NP (green fluorescence) intensity increased at 60 and 120 min after virus adsorption, whereas no increase was observed under NH_4_Cl treatment (Fig. 5a). These findings confirmed that the ICV uncoating process can be evaluated using this experimental system.

Next, we conducted a similar analysis using recombinant viruses harbouring mutant CM2. We found that the nuclear fluorescence intensity of NPs in cells infected with rCM2-Ala47–48 and rCM2-Ala67–69 viruses was comparable to that of rWT. However, in cells infected with rCM2-Ala73–75 and rCM2-Ala85–87 viruses, the nuclear NP fluorescence intensity at 60 and 120 min after adsorption was significantly reduced compared with that of rWT (Figs 5b and S1). These results suggested that uncoating is impaired and the nuclear transport of the viral genome is reduced in rCM2-Ala73–75- and rCM2-Ala85–87-infected cells.

### Functional analysis of the sequence at CM2 113–115 using VLPs

**Fig. 6.**
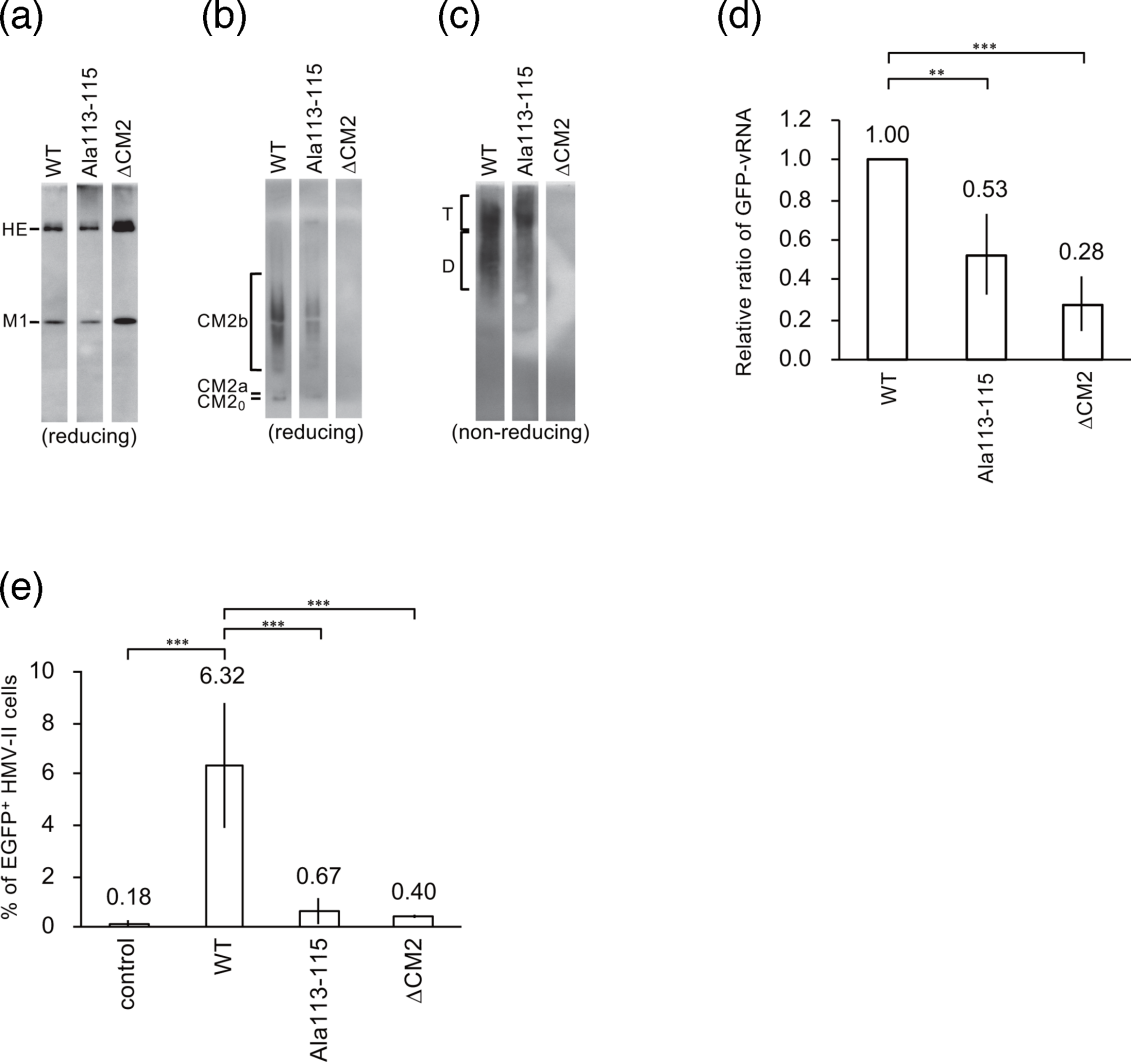
Characterization of CM2 Ala113–115 mutant VLPs. Western blot analysis of HE and M1 protein (**a**), CM2 protein (**b**) under reducing conditions and CM2 proteins under non-reducing condition (**c**) in VLPs. (**d**) Comparison of GFP-vRNA levels in VLPs. The amount of GFP-vRNA in each purified VLP was measured by real-time RT-PCR and quantified using WT-VLP as a normalization standard. (**e**) Analysis of GFP expression in VLP-infected cells. HMV-II cells were infected with each VLP, and the percentage of GFP-positive cells was analysed by flow cytometry after 48 h of incubation. Control indicates the background level in cells without VLP infection. Statistical analysis was performed using one-way ANOVA followed by Tukey’s post-hoc test (*P<0.05, **P<0.01 and ***P<0.001).

As described above, we attempted to generate the rCM2-Ala113–115 virus but were unable to recover it. These results suggested that the 113–115 amino acid sequence of CM2 is essential for viral replication. Therefore, to investigate the role of this sequence in viral replication, we generated ICV-like particles (VLPs) carrying the GFP gene (GFP-vRNA) in their genome and examined their involvement in virion formation, genome packaging and uncoating. First, we generated WT-VLPs, VLPs with CM2-Ala113–115 protein (CM2-Ala113–115-VLPs) and CM2-deficient VLPs (∆CM2-VLPs). To compare the production of these VLPs, we measured the haemagglutination (HA) titre of the culture supernatant, total protein concentration of purified VLPs and expression level of the HE protein in VLPs. All values were comparable among the three VLPs (data not shown). Western blot analysis showed modest elevations in HE and M1 protein levels in ΔCM2-VLPs relative to WT-VLPs, whereas levels in CM2-Ala113–115-VLPs remained largely unchanged (Fig. 6a). These results suggested that the CM2-Ala113–115 mutation had no significant effect on VLP production or structural protein expression. Although stronger HE and M1 bands were detected in ΔCM2-VLPs, this is not inconsistent with the other indicators of VLP production such as HA titres and total protein levels. Therefore, we consider that this difference does not influence the interpretation of the results. CM2 protein levels in VLPs revealed that CM2 protein levels were lower in CM2-Ala113–115-VLPs than in WT-VLPs (Fig. 6b). Western blot analysis of CM2 multimerization in VLPs under non-reducing conditions showed that the proportion of dimers in CM2-Ala113–115-VLPs was lower than that in WT-VLPs; however, we observed tetramer formation (Fig. 6c), suggesting that this amino acid sequence mutation does not impair CM2 oligomerization.

Next, to analyse the effect of amino acid residues 113–115 on viral genome packaging, we quantified the amount of GFP-vRNA via real-time RT-PCR using RNA extracted from purified VLPs. The amount of GFP-vRNA was 53 and 28% of that in WT-VLPs, respectively (Fig. 6d). These results suggested that the genomic packaging of CM2-Ala113–115-VLPs is less efficient than that of WT-VLPs.

Furthermore, to investigate the effect of the amino acid sequence at positions 113–115 of CM2 on the uncoating process, we examined the reporter gene expression in VLP-infected cells. In this experiment, HMV-II cells were inoculated with the same amount of VLP and then superinfected with WT helper virus. This induced the expression of the GFP protein derived from GFP-vRNA, which was transferred into the nucleus of VLP-infected cells. The GFP fluorescence intensity, measured by flow cytometry, was used as an indicator of the amount of GFP-vRNA that migrated into the nucleus. The population of GFP-positive cells was 6.32±2.43% for WT-VLPs, 0.67±0.50% for CM2-Ala113–115-VLPs and 0.40±0.04% for ∆CM2-VLPs, indicating that GFP expression in CM2-Ala113–115-VLP-infected cells was reduced to a level comparable to that of ∆CM2-VLPs (Fig. 6e). These results suggested that the amino acid sequences at positions 113–115 of CM2 are required for viral uncoating.

## Discussion

In this study, we demonstrated that the cytoplasmic region of CM2 plays an important role in ICV proliferation. The results are summarized in Table 1 and evaluated from the perspectives of viral replication, protein expression, genome packaging and uncoating. Recombinant viruses harbouring mutations at amino acid positions 67–69, 73–75 and 85–87 in CM2 exhibited reduced replication efficiencies (Fig. 1b). In these mutant viruses, CM2 protein levels in both virions and infected cells were significantly reduced compared with those in rWT viruses (Figs.2  4). Furthermore, a similar reduction in CM2 protein levels was observed in CM2-Ala113-115-VLPs, consistent with previous reports [15]. These results suggested that multiple sequences in the cytoplasmic region of the CM2 protein are involved in its intracellular stability and contribute to its efficient incorporation into virions.

**Table 1.** Effects of amino acid mutations in the cytoplasmic region of CM2 on viral replication and protein expression

Virus or VLP	Replication	Amount of CM2 protein			Amount of vRNA	Uncoating
		Virion	Intracellular	Cell surface		
rCM2-Ala47–48	nc	nc	nc	nc	nc	nc
rCM2-Ala67–69	Reduced	Reduced	Reduced	nc	Reduced	nc
rCM2-Ala73–75	Reduced	Reduced	Reduced	Reduced	Reduced	Reduced
rCM2-Ala85–87	Reduced	Reduced	Reduced	nc	Reduced	Reduced
CM2-Ala113–115-VLP	nd	Reduced	Reduced*	nd	Reduced	Reduced

*Data cited from reference [14].

nc, no change; nd, not determined.

Next, we analysed CM2 protein levels on the cell surface and found that surface expression was significantly reduced in rCM2-Ala73–75-infected cells compared with that in rWT-infected cells (Fig. 4b, d). In contrast, rCM2-Ala85–87 virus showed no significant difference in the amount of CM2 protein on the cell surface, although the amount of CM2 protein in infected cells was reduced (Fig. 4). These results suggested that the amino acid sequence at positions 73–75 is involved in CM2 protein localization to the cell surface, whereas the sequence at positions 85–87 may be involved in different mechanisms, such as subcellular localization or protein transport. Additionally, cells infected with CM2-Ala67–69 and CM2-Ala73–75 mutant viruses exhibited elevated HE protein levels, even though CM2 expression was diminished (Fig. 4a, b). These findings imply that defects in virion budding or disrupted CM2–HE interactions may contribute to enhanced intracellular retention of HE. Further investigation is warranted to clarify the underlying mechanisms.

In rCM2-Ala73–75, 85–87 viruses and CM2-Ala113–115-VLPs, the amount of vRNA transferred to the nucleus during the early stage of viral infection was reduced compared with that in rWT and WT-VLPs (Figs5  6e). Previous studies have shown that CM2 proteins are involved in proton ion permeability and uncoating [9,27]. Collectively, we hypothesized that these mutations reduced the amount of CM2 protein in viral particles, thereby reducing proton permeability and resulting in inefficient uncoating. In particular, the CM2-Ala113–115 mutation suppressed uncoating to a level comparable to that of the background, which may have resulted in difficulties in virus recovery.

In addition, the C-terminal portion (residues 111–115) of the CM2 cytoplasmic region is composed of hydrophobic amino acids. A previous study reported [28] that a mutation of the hydrophobic motif at positions 91–94 in the M2 protein of IAV causes protein conformation alterations. Thus, the CM2-Ala113–115 mutation may affect the structure of CM2, thereby impairing proton ion permeability and replication. Furthermore, genomic packaging was reduced to ~50% of WT in this mutant VLP, suggesting that this region may be involved not only in uncoating but also in genomic packaging. In influenza A and B viruses, the terminal coding regions of individual gene segments contribute to selective genome packaging [29,30]. Given that residues 113–115 of CM2 align with the 3′ end of the M gene coding region, mutations at these sites may influence both CM2 protein function and the packaging efficiency of M-segment vRNA. The possible influence of RNA sequences on genome packaging needs to be assessed through additional research using GFP-vRNA-based VLP systems.

We previously reported that the amino acid sequence at positions 47–48 of the CM2 protein is involved in N-glycosylation and that the sequence at positions 73–75 is involved in N-glycosylation and tetramer formation [15]. However, in the present study, no clear differences were observed in the N-glycosylation or oligomerization of the CM2 protein in virions harbouring these mutations (Figs2a  3). This discrepancy may be attributed to the selective incorporation of CM2 proteins with proper post-translational modifications into virions, even though the overall amount of post-translationally modified CM2 was reduced in cells expressing these mutant proteins. These results were consistent with previous reports, indicating that glycosylation and oligomerization are important for ICV replication [12,13].

Our findings suggested that the cytoplasmic region of CM2 plays a multifaceted role in ICV replication. Specific amino acid residues (67–69, 73–75, 85–87 and 113–115) appear to be involved in intracellular stability, localization, incorporation into viral particles, uncoating and viral genome packaging, and different mechanisms may be involved in each region. These insights advance our structural and functional understanding of CM2 and may also provide implications for studies of viral membrane proteins in other viruses.

## Supplementary material

10.1099/jgv.0.002165 Uncited Fig. S1.

10.1099/jgv.0.002165 Uncited Table S1.

## Abbreviations

HA: haemagglutination
IAV: influenza A virus
ICV: influenza C virus
MEM: minimal essential medium
MOI: multiplicity of infection
RT-PCR: reverse transcription PCR
TPCK: tosylamide phenylmethyl chloromethyl ketone
VLPs: virus-like particles
vRNA: viral RNA
