# Supplementary material for: Cytoplasmic domain of CM2 is involved in the replication of influenza C virus
Source: J Gen Virol. 2025 Oct 17;106(10):002165. doi: 10.1099/jgv.0.002165 (PMC12534170; doi:10.1099/jgv.0.002165)
Supplement: Uncited Fig. S1. [file jgv-106-02165-s001.pdf]

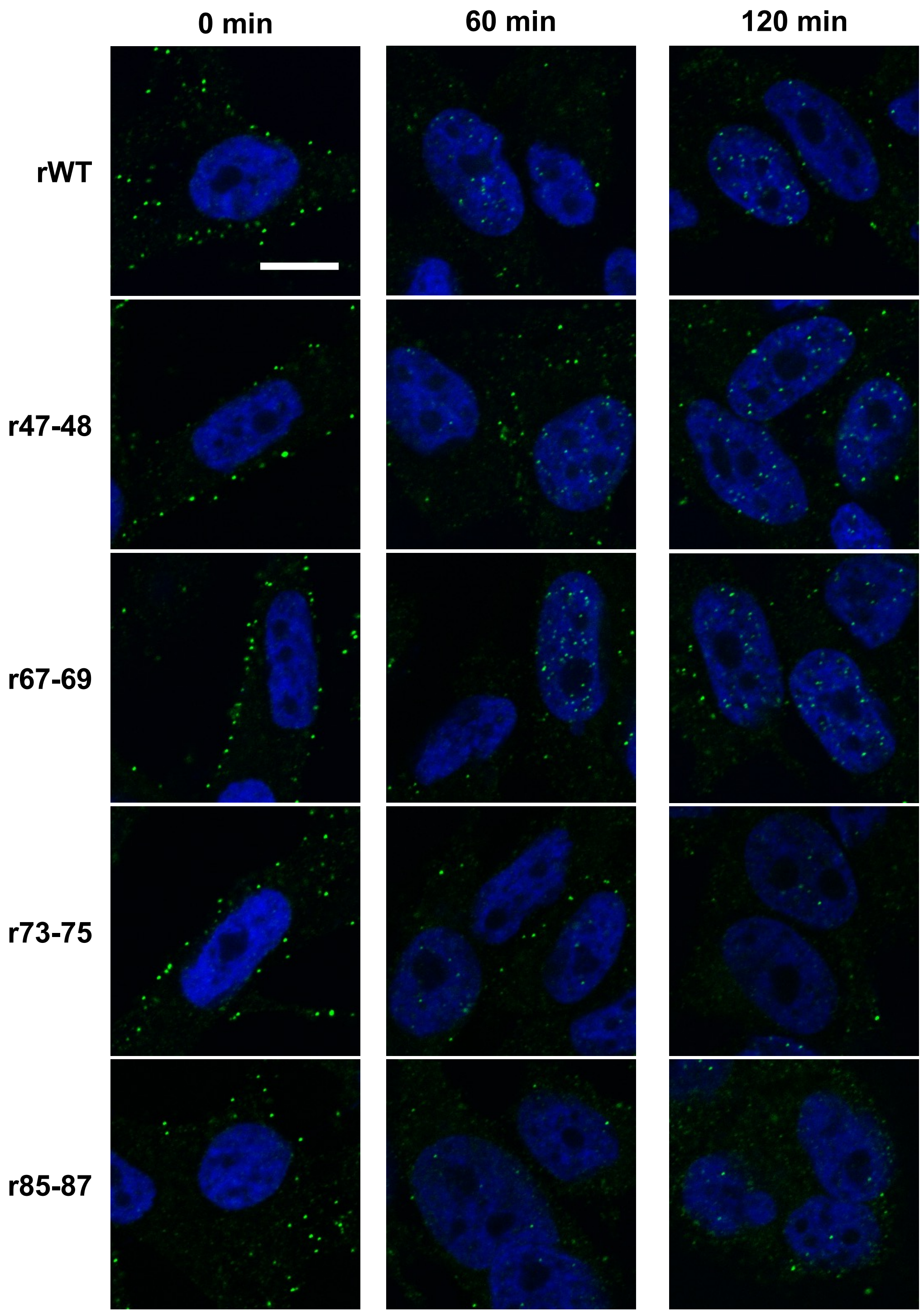

Figure S1. Fluorescence microscopy images showing the intracellular localization of vRNPs (NPs) during the early stage of viral infection. HMV-II cells were infected with rWT, rCM2-Ala47-48, rCM2-Ala67-69, rCM2-Ala73-75, or rCM2-Ala85-87 viruses, and observed immediately after incubation at 34°C or after 60 and 120 min of incubation. NPs were stained with green fluorescence, and nuclei with blue fluorescence. Scale bars indicate 10 μm.
