# Supplementary material for: Cytoplasmic domain of CM2 is involved in the replication of influenza C virus
Source: J Gen Virol. 2025 Oct 17;106(10):002165. doi: 10.1099/jgv.0.002165 (PMC12534170; doi:10.1099/jgv.0.002165)
Supplement: Uncited Table S1. [file jgv-106-02165-s002.pdf]

Table S1. Nucleotide sequences of primers used for the generation of mutant viruses.

	Sequences (5' to 3')
CM2 Ala (47-48) F	GCAGCCATAATTGAACTTGTCAATGGT
CM2 Ala (47-48) R	TACTAATAAATACAACATAG
CM2 Ala (67-69) F	GCTCGAGCCACCACAATTATGCCTGAAAT
CM2 Ala (67-69) R	TCCACACCATCTCTCCCATC
CM2 Ala (73-75) F	GCGGCTGCAATTGACTCGATGGAAAAAGAT
CM2 Ala (73-75) R	AATTGTGGTCTTTATATCTC
CM2 Ala (85-87) F	GCTGCTGCGGAGAGACTTGACCTGGGAGAGGATGCT
CM2 Ala (85-87) R	GGCAATATCTTTTTCCATCGAG
polI-CM2-Ala113-115F	GCTGCAGCTTAATTACCTTG
CM2 Ala (113-115) R	AATACCATCATTGGAAAAAG
